# Tn-Seq reveals hidden complexity in the utilization of host-derived glutathione in *Francisella tularensis*

**DOI:** 10.1371/journal.ppat.1008566

**Published:** 2020-06-03

**Authors:** Kathryn M. Ramsey, Hannah E. Ledvina, Tenayaann M. Tresko, Jamie M. Wandzilak, Catherine A. Tower, Thomas Tallo, Caroline E. Schramm, S. Brook Peterson, Shawn J. Skerrett, Joseph D. Mougous, Simon L. Dove

**Affiliations:** 1 Division of Infectious Diseases, Boston Children’s Hospital, Harvard Medical School, Boston, Massachusetts, United States of America; 2 Departments of Cell and Molecular Biology and Biomedical and Pharmaceutical Sciences, University of Rhode Island, Kingston, Rhode Island, United States of America; 3 Department of Microbiology, University of Washington School of Medicine, Seattle, Washington, United States of America; 4 Division of Pulmonary, Critical Care and Sleep Medicine, Harborview Medical Center, University of Washington, Seattle, Washington, United States of America; 5 Howard Hughes Medical Institute, University of Washington, Seattle, Washington, United States of America; Stanford University School of Medicine, UNITED STATES

## Abstract

Host-derived glutathione (GSH) is an essential source of cysteine for the intracellular pathogen *Francisella tularensis*. In a comprehensive transposon insertion sequencing screen, we identified several *F*. *tularensis* genes that play central and previously unappreciated roles in the utilization of GSH during the growth of the bacterium in macrophages. We show that one of these, a gene we named *dptA*, encodes a proton-dependent oligopeptide transporter that enables growth of the organism on the dipeptide Cys-Gly, a key breakdown product of GSH generated by the enzyme γ-glutamyltranspeptidase (GGT). Although GGT was thought to be the principal enzyme involved in GSH breakdown in *F*. *tularensis*, our screen identified a second enzyme, referred to as ChaC, that is also involved in the utilization of exogenous GSH. However, unlike GGT and DptA, we show that the importance of ChaC in supporting intramacrophage growth extends beyond cysteine acquisition. Taken together, our findings provide a compendium of *F*. *tularensis* genes required for intracellular growth and identify new players in the metabolism of GSH that could be attractive targets for therapeutic intervention.

## Author summary

Prominent amongst the host-derived nutrients *Francisella tularensis* requires for intracellular growth is glutathione (GSH), a tripeptide which serves as an essential source of cysteine. Here we comprehensively identify those genes *F*. *tularensis* requires for intramacrophage growth and characterize two that play previously unrecognized roles in GSH utilization. One of these encodes a transporter of the dipeptide Cys-Gly which is a breakdown product of GSH, while the other encodes a member of the ChaC family of proteins which we show plays a role in GSH breakdown. Our findings uncover a critical role for a member of the proton-dependent oligopeptide transporter family in *Francisella* intramacrophage growth and provide the first example of a ChaC family enzyme acting to catabolize GSH in bacteria.

## Introduction

*Francisella tularensis* is a facultative intracellular pathogen and the etiological agent of the disease tularemia, which can be fatal if left untreated [1]. Four subspecies of *F*. *tularensis* have been recognized (*tularensis*, *holarctica*, *mediasiatica* and *novicida*) which possess differential host ranges and cause disease with varying degrees of morbidity and mortality [2, 3]. While *F*. *tularensis* is capable of replicating in a variety of cell types, growth within macrophages is believed to be the primary mediator of disease [4, 5]. However, the precise virulence mechanisms employed for intracellular proliferation are largely uncharacterized.

*F*. *tularensis* initially enters cells through the endosomal pathway but must escape this degradative compartment to replicate within the cytoplasm. Perhaps the best characterized virulence factor utilized by *F*. *tularensis* is the type VI secretion system subtype 2 (T6SS^ii^) pathway encoded by genes present on the so-called *Francisella* pathogenicity island (FPI) [6, 7]. This system has been demonstrated to secrete a set of effector proteins which mediate escape into the cytoplasm [8–10]. Additional factors known to be important for growth in host cells include many metabolic pathways, including those involved in purine biosynthesis and branched-amino acid utilization (reviewed in [11, 12]).

One host-derived metabolite that is essential for the intramacrophage growth of *Francisella* is the low molecular weight thiol glutathione (GSH; γGlu-Cys-Gly). *Francisella* is a cysteine auxotroph and GSH is a necessary source of cysteine for intracellular replication [13]. This molecule is found at high concentrations (1–10 mM) in the cytoplasm of macrophages, as well as many other eukaryotic cells [14]. Existing literature suggests that the principal enzyme responsible for initiating degradation of GSH in *Francisella* is γ-glutamyl transpeptidase (GGT), which cleaves this tripeptide into glutamate and the dipeptide Cys-Gly [13, 15]. However, this is only the presumed first step of GSH utilization and the role of other factors that work in concert with GGT to facilitate GSH utilization has not been addressed. Moreover, *Francisella* encodes a member of the ChaC family of proteins which have been shown to contribute to the breakdown of GSH in eukaryotes [16–18]. Whether *Francisella* employs enzymes that act in parallel with GGT to breakdown GSH was not known.

While several screens have been employed to identify genes important for the intracellular replication of multiple *F*. *tularensis* subspecies [19–30], a comprehensive description of genes that are specifically required for the intramacrophage growth of this organism has been lacking. Here, we used transposon insertion sequencing (Tn-Seq) to identify the genes that are required for the intracellular growth of the live vaccine strain of *F*. *tularensis* (LVS). We leveraged the hits of our screen to define a unique pathway for the catabolism of GSH in *Francisella*. This pathway includes a promiscuous proton-dependent dipeptide transporter responsible for the import of Cys–Gly and a ChaC-family enzyme that can function alongside GGT to scavenge cysteine from GSH. Our work reveals unexpected complexity in the utilization of GSH by *Francisella* and provides a comprehensive catalog of genes required for the intracellular growth of this pathogen.

## Results

### Characterization of a highly saturated transposon insertion library for Tn-Seq

The ability of *F*. *tularensis* to cause disease is dependent principally upon its ability to grow within macrophages (reviewed in [3, 31, 32]). Although a number of genetic screens have been performed in several subspecies to identity genes required for this function [19, 21, 24, 25, 28], a consensus as to the bacterial requirements for intramacrophage growth remains lacking. To comprehensively identify *Francisella* genes critical for growth and survival within macrophages, as well as for growth *in vitro*, we created a saturated library of transposon insertion mutants in the live vaccine strain of *F*. *tularensis* (LVS) for use in transposon insertion sequencing (Tn-Seq) experiments. In order to use the insertion sequencing (INSeq) method of transposon insertion identification [33], we modified a previously used mariner transposon, which specifically inserts into TA dinucleotides [34]. Using the modified transposon encoding kanamycin resistance, we mutagenized LVS six independent times and combined approximately 800,000 kanamycin-resistant colonies into a single library.

**Fig 1:**
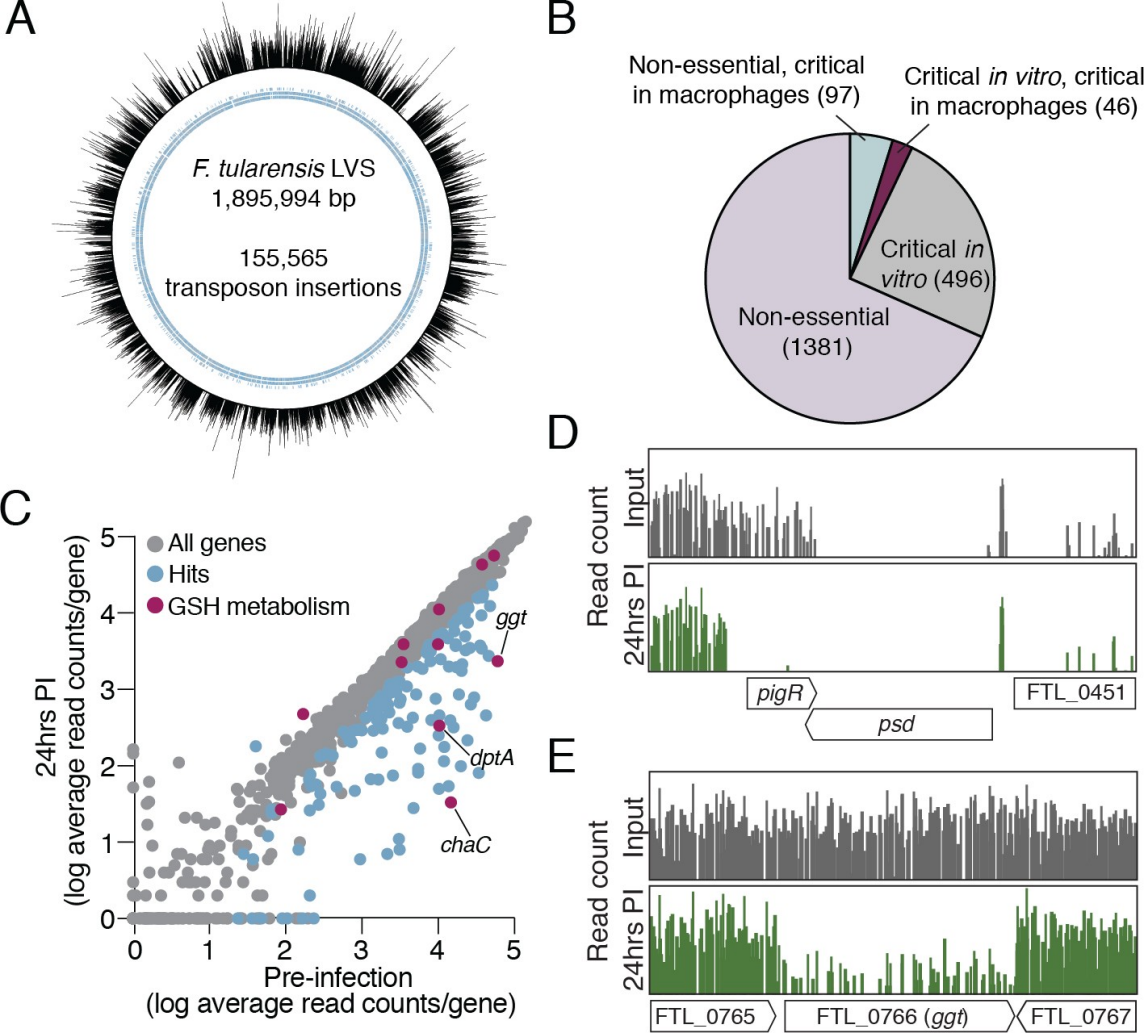
Highly saturated Tn-Seq screen in *F*. *tularensis* LVS to identify genes required for intramacrophage growth. (A) Map of transposon insertions across the *F*. *tularensis* genome. Of all the potential transposon insertion sites (TA dinucleotides) genome-wide, 75% contain a transposon insertion. Each line corresponds to one mutant with a single insertion; the line height corresponds to the relative abundance of sequencing reads identified for each individual insertion. Blue inner circle represents genes. (B) EL-ARTIST and Con-ARTIST analysis of Tn-Seq screen reveals genes that are non-essential under any tested condition (lavender), critical *in vitro* (grey), critical *in vitro* and in macrophages (maroon), and those that are non-essential *in vitro* but critical in macrophages (blue). (C) The relative abundance of mutations in each gene (average read count per gene) in the mutant pool used to infect J774A.1 cells (pre-infection) and in mutants recovered after 24 hours of infection. Genes significantly more or less abundant after infection (at least 2-fold changed, with 4 or more informative insertion sites, p < 0.05) are colored in blue. Genes involved in GSH metabolism are highlighted in purple. (D-E) Transposon insertion profiles for the indicated genetic regions from the input library (grey) and 24 hr post infection (green). Line height represents the relative abundance of sequencing reads at that position on a log scale.

We determined which genes are critical for *in vitro* growth by growing cells of the transposon mutant library on solid media and using extracted genomic DNA to specifically amplify bacterial transposon-chromosome junctions. Through this approach we identified 155,565 unique insertion events in our mutant library, corresponding to 74.6% of all the potential TA insertion sites (208,625) and one insertion event approximately every 12 bp (Fig 1A). To assess saturation of the mutant library at the individual gene level, we determined the percentage of TA sites in each gene with identified insertions. About 20% of genes had insertions in fewer than 10% of TA sites, suggesting these genes are essential under these growth conditions. In contrast, we could identify insertions in 90% or more of the TA sites for the majority of genes (73%), as would be expected for nonessential genes. The strong bimodal distribution of the percentage of TA sites disrupted per gene indicates that the library is highly saturated, as this distribution provides evidence that TA sites only lack an insertion when a transposon at that site would disrupt an essential function (S1B Fig). In comparison, the prior most saturated transposon library used to screen for genes important for the intracellular replication of *Francisella* contained ~15,000 mutant clones (approximately one-tenth of the number of unique insertions in our mutant library) and use of the transposon site hybridization (TraSH) methodology precluded an assessment of the total number of unique insertions [24]. We used ARTIST, which determines essential genetic regions using a hidden Markov model [35], to identify 481 essential genes as well as 61 genes specifying proteins with both essential and non-essential domains (S1A Fig and S1 Table).

### Tn-Seq identifies genes and pathways critical for intramacrophage replication

In order to define LVS genes critical for intramacrophage growth, we infected J774A.1 murine macrophage-like cells with our transposon mutant library (with macrophages in ~80-fold excess of the complexity of our mutant library). After twenty-four hours, macrophages were lysed, and bacterial genomic DNA was extracted for library construction. Using Con-ARTIST [35] to compare a sample of the inoculum to the mutants that survived within macrophages, we identified 142 genes that were important for intramacrophage survival (Fig 1B and 1C and S2 Table).

Several lines of evidence indicate that our Tn-Seq-based screen for genes important for intramacrophage growth was unbiased and comprehensive. With regard to bias, if our infection conditions, isolation of bacterial genomic DNA from macrophages, or sequencing depth of recovered bacterial DNA generated a bottleneck, we would expect stochastic loss of transposon insertions in genes non-essential for intramacrophage growth. However, we did not identify a shift in insertion saturation in non-essential genes following growth of our mutant library in macrophages, strongly suggesting the lack of a bottleneck (S1B Fig). Instead, the TA insertion saturation remained strongly bimodal, consistent with recovery of essentially all mutants that were not defective for growth in macrophage. The comprehensive nature of our screen is highlighted by our identification of all thirteen genes encoding purine biosynthesis enzymes as either critical for survival *in vitro* or critical for growth or survival within macrophages (S2 Fig). Purine biosynthesis is critical for *F*. *tularensis* intracellular survival, yet prior screens had individually identified only up to eight genes in this pathway as important for the process [19–30]. In addition, the gene encoding PigR, a transcription regulator critical for intramacrophage growth and virulence [34], was a strong hit in our screen despite its small size (336 bp) and previous lack of identification in other unbiased screens for factors important for intramacrophage growth or virulence (Fig 1D, S2 Table). Finally, our screen identified ~68% of the genes previously ascribed importance for growth in the J774 murine macrophage cell line [21, 25] while also revealing 89 additional genes as being critical for this process.

The ability of *F*. *tularensis* to grow within macrophages is dependent upon several nutrients that are derived from the host [36–39]. Prominent amongst these is GSH, a tripeptide whose catabolism provides a source of cysteine that *F*. *tularensis* requires for growth [13]. Consistent with previous findings, our screen identified *ggt*, which encodes a γ-glutamyl transpeptidase, as important for intramacrophage growth (Fig 1C and 1E and S2 Table) [13]. The GGT enzyme mediates the first step in the breakdown of GSH, generating glutamate and the dipeptide Cys-Gly [40]. The *F*. *tularensis* enzyme contains a strongly predicted Sec signal sequence, suggesting that it localizes to the periplasm (S3 Fig). We therefore reasoned that utilization of Cys-Gly as a source of cysteine may require the action of a transporter that shuttles Cys-Gly from the periplasm to the bacterial cytoplasm where Cys-Gly could be broken down further to individual amino acid components. Moreover, we reasoned that the gene specifying a Cys-Gly peptide transporter would be amongst those we identified as essential for intramacrophage growth using Tn-Seq (S2 Table).

### Identification of a putative Cys-Gly transporter

**Fig 2:**
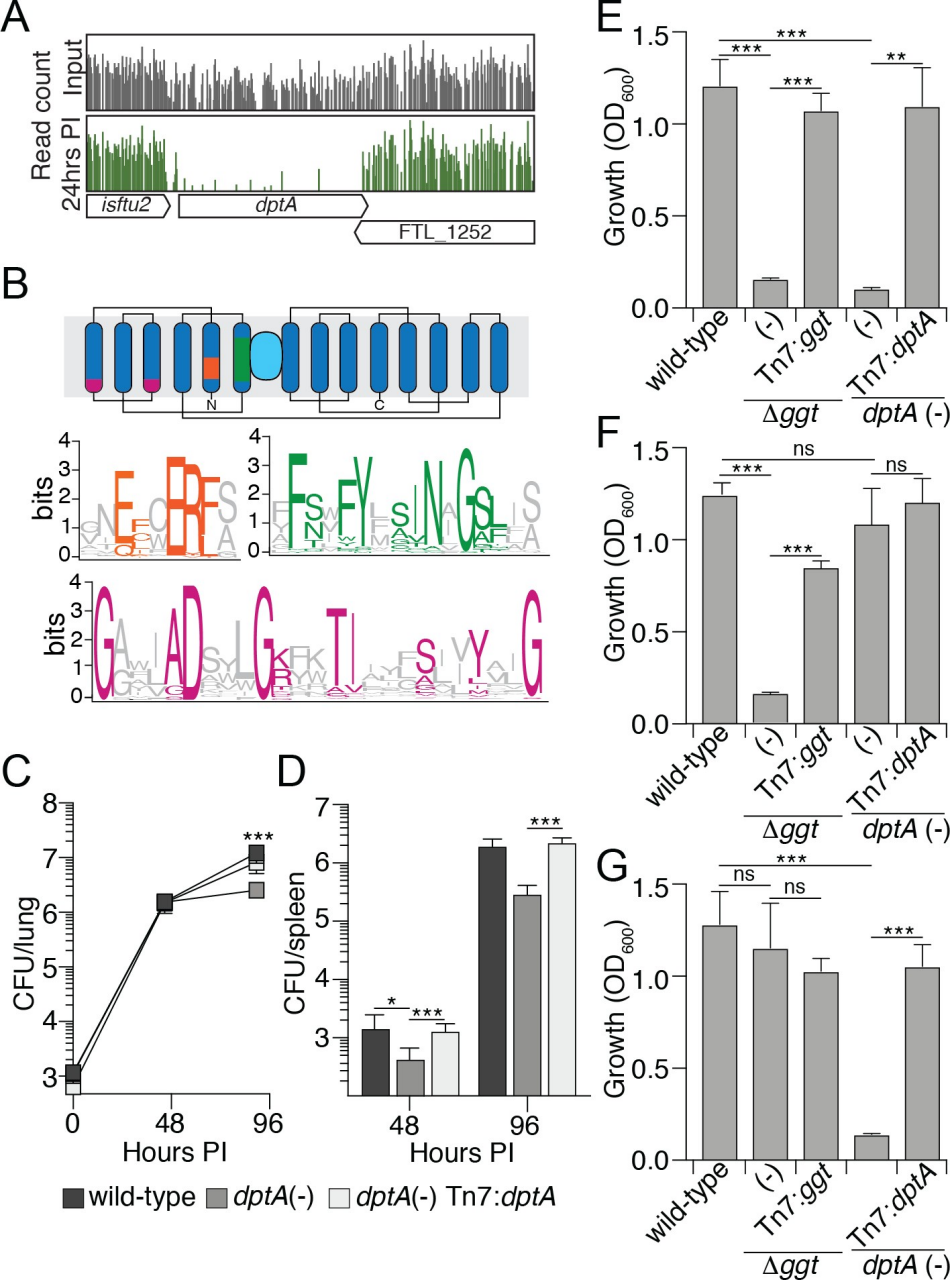
DptA is a POT that contributes to GSH utilization by LVS. (A) Transposon insertion profiles for the genetic region encompassing *dptA* from the input library (grey) and 24 hr post infection (green). Line height represents the relative abundance of sequencing reads at that position on a log scale. (B) Graphical representation of the predicted topology of DptA with conserved sequences motifs found in POT proteins highlighted (proton binding motif, orange; PTR2 domain 1, green; PTR2 domain 2, pink; peptide binding region, light blue). Sequence logo indicates motifs conserved in the POT family derived from an alignment of representative POT proteins. (C) Quantification of the bacterial burdens in the lungs of mice infected via aerosolization with the indicated strains of LVS. (D) Quantification of the bacterial dissemination to, and burdens within the spleens of mice infected via aerosolization with the indicated strains of LVS. (E-G) OD_600_ measurements of the indicated strains of LVS after 16 hrs of growth in CDM with glutathione (E), γGlu-Cys (F) or Cys-Gly (G). Data in (C-G) are shown as mean ± s.d. Asterisks represent statistically significant differences (Student’s t test; ***p≤0.0005, **p≤0.005, *p≤0.05, ns, not significant).

A top hit from our *in vivo* Tn-Seq screen was FTL_1251, which encodes a putative proton-dependent oligopeptide transporter (POT) (Figs 1C and 2A and S2 Table). This group of transporters are part of the major facilitator superfamily and are characterized by the presence of 12–14 transmembrane domains, a signature motif of charged proton-binding residues within a transmembrane domain (ExxERF), and two PTR2 domains [41]. Each of these motifs was identified in FTL_1251 (residues 24–32, 72–96, and 153–167, respectively) and this protein contains 14 predicted transmembrane domains (Fig 2B). POT proteins often transport multiple peptides across the inner membrane, though these are generally related in sequence and chemical properties [42–44]. LVS is predicted to encode eight POT proteins; however, only FTL_1251, henceforth referred to as DptA, was hit in our screen (S2 Table). As no other putative peptide transporters were hit in our screen we hypothesized DptA contributes to intracellular growth of *F*. *tularanesis* by acting within the GGT pathway for GSH catabolism.

As a first step toward characterizing DptA, we probed its contribution to colonization of mice following infection by the aerosol route. Consistent with our Tn-Seq screen, disruption of *dptA* resulted in significantly reduced proliferation within the lungs, as well as lowered dissemination to distal organs (Fig 2C and 2D). The observed *in vivo* defects could be complemented by ectopic expression of *dptA* from the Tn7 attachment site on the LVS chromosome. With the confirmation that the predicted transporter contributes to virulence, we set out to determine if DptA plays a role in GSH metabolism.

To evaluate the contribution of DptA to GSH metabolism, we measured the growth of a LVS *dptA*(-) strain in Chamberlain’s defined media (CDM) containing a panel of cysteine-containing peptides. We also tested a strain of LVS in which we deleted the gene encoding GGT (LVS Δ*ggt*), which was previously shown to be essential for the growth of LVS with GSH provided as a sole source of cysteine [13]. Indeed, we found that in LVS, GGT is required for growth on GSH and γGlu-Cys, but not on cysteine or Cys-Gly (Fig 2E–2G and S4A and S4B Fig). Moreover, as expected, the *in vitro* growth defects of the LVS Δ*ggt* mutant strain in media supplemented with either GSH or γGlu-Cys could be complemented by ectopic expression of *ggt* from the Tn7 attachment site on the LVS chromosome (Fig 2E–2F). On the contrary, cells lacking DptA show no detectable growth on GSH or Cys-Gly and proliferate similarly to cells of the wild-type strain in the presence of γGlu-Cys or cysteine (Fig 2E–2G and S4A and S4B Fig). Furthermore, the *in vitro* growth defects of the LVS *dptA* mutant strain in media supplemented with either GSH or Cys-Gly could be complemented by ectopic expression of *dptA* from the Tn7 attachment site (Fig 2E and 2G and S4A and S4B Fig). These data suggest that DptA is required for the transport of Cys-Gly. Further, they suggest that under the conditions of our *in vitro* growth assays, GGT and DptA constitute the single pathway for GSH degradation as well as transport of the resulting Cys-Gly breakdown product.

### DptA is a promiscuous transporter that aids in cysteine acquisition during intramacrophage growth

**Fig 3:**
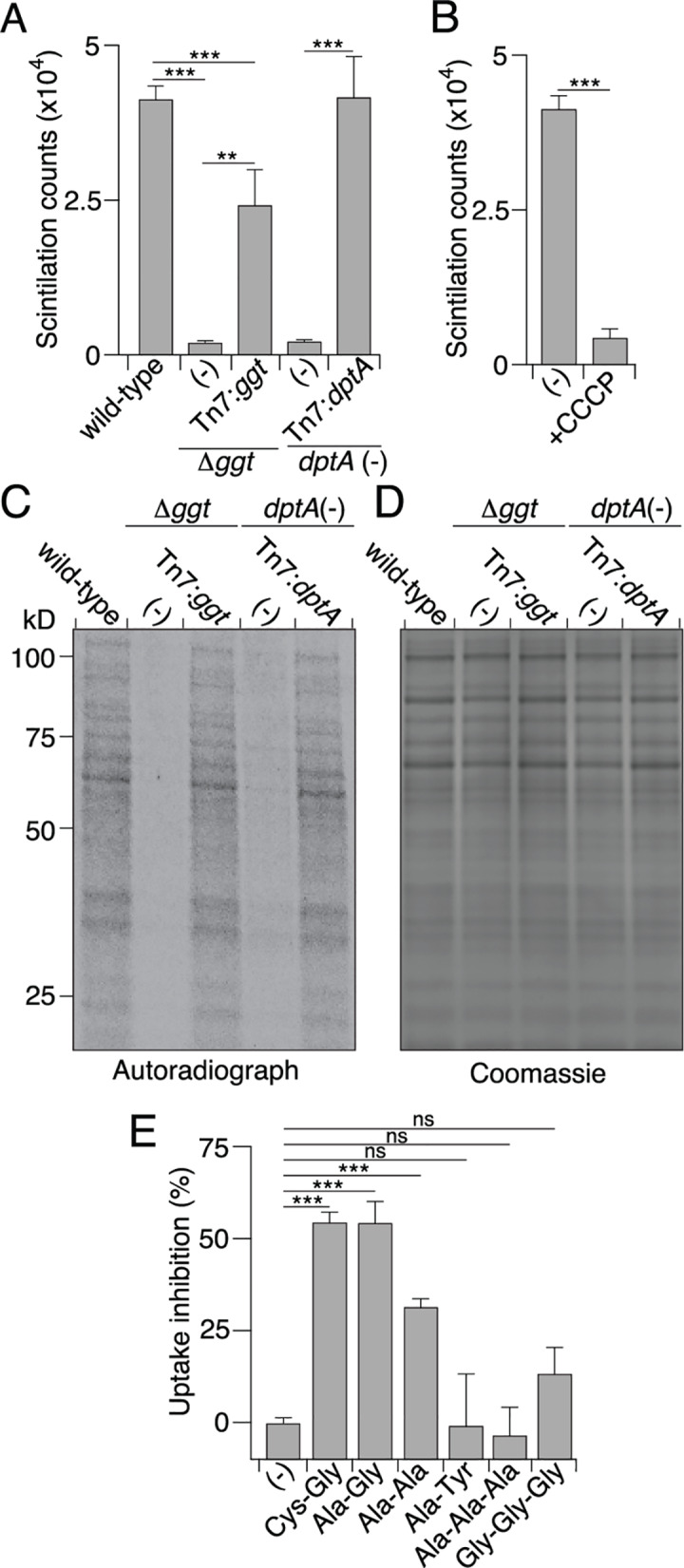
DptA is the sole Cys-Gly transporter in LVS. (A-B) Quantification of the intracellular levels of ^3^H-GSH byproducts after 45 min incubation with the labeled substrate in the indicated strains of LVS (A) or WT LVS with and without CCCP (B). (C) Autoradiograph of SDS-PAGE separated proteins produced by growing the indicated strains of LVS in CDM lacking cysteine supplemented with ^35^S-Cysteine containing GSH. (D) Coomassie-blue staining of proteins generated as described in (C). (E) Percent of ^3^H-GSH uptake that was inhibited by addition 100 μM of the indicated di- or tripeptides. Data in (A, B and E) are shown as the mean ± s.d. Asterisks represent statistically significant differences (Student’s t test; ***p≤0.0005, **p≤0.005, ns, not significant).

To more definitively characterize the role of DptA in LVS, we turned to assays measuring the cellular uptake of radiolabeled GSH (^3^H-glycine GSH (^3^H-GSH)). Cells of the LVS Δ*ggt* mutant strain unable to break down GSH displayed low levels of intracellular radiolabel, consistent with previous findings that LVS is unable to harness GSH as a source of cysteine (Fig 3A) [13]. Disruption of *dptA* likewise dramatically reduced intracellular radiolabel accumulation, supporting our hypothesis that DptA transports Cys-Gly, the product of GGT activity on GSH. Treatment of wild-type cells with CCCP, which dissipates the proton motive force, inhibited accumulation to the levels of the *dptA* mutant strain, in-line with our assignment of DptA to the POT family (Fig 3B). Moreover, this result suggests that DptA accounts for all detectable proton motive force-driven transport of GSH derivatives. Additional metabolic labeling experiments using ^35^S-cysteine-containing GSH confirmed that GSH-derived cysteine is utilized for protein biogenesis (Fig 3C and 3D).

We next sought to determine the specificity of DptA. Competition for the uptake of ^3^H-GSH-derived species by various di- and tri-peptides revealed that dipeptides with a C-terminal Gly are preferred substrates. Dipeptides with small, nonpolar C-terminal residues and a tripeptide composed entirely of Gly displayed detectable competition with ^3^H-GSH derivatives; however, their efficiency was significantly lower relative to Cys-Gly. Other tripeptides and dipeptides containing a bulky C-terminal residue were unable to compete with ^3^H-GSH products for uptake at the concentrations tested (Fig 3E). These results support our hypothesis that DptA is a key transporter of the Cys-Gly dipeptide in LVS.

**Fig 4:**
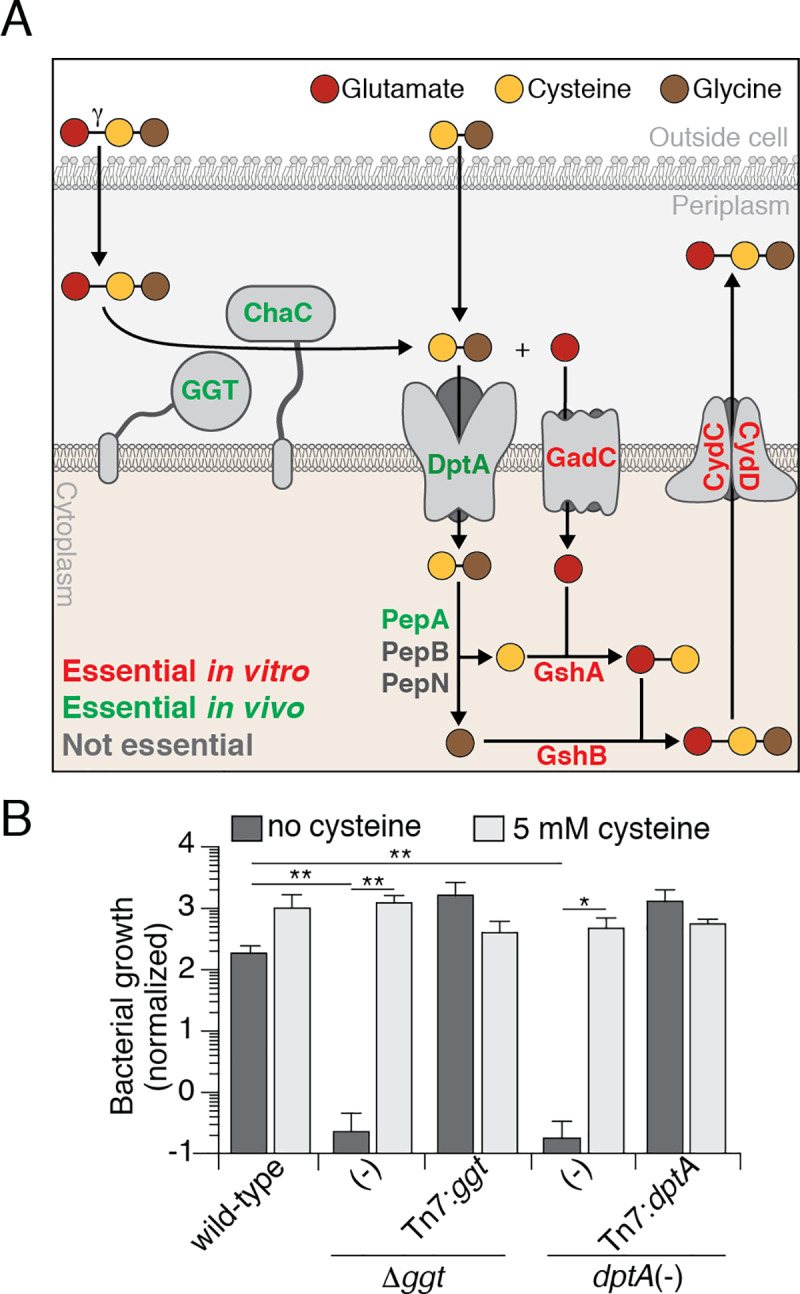
DptA and GGT are essential for *Francisella* growth *in vivo* in the absence of exogenous cysteine. (A) Model depicting the localization and role of the indicated enzymes and transporters in the metabolism of GSH by *F*. *tularensis*. The color of the name text indicates the essentiality of that protein based on our Tn-Seq screen in J774 macrophages; essential for growth in MH broth, red; essential for growth in J774 macrophages, green; non-essential in the tested conditions, grey. (B) Growth of the indicated strains of LVS in J774 cells, normalized to bacterial counts at 2 hrs post infection. J774 cells were either left untreated or treated with 5 mM cysteine prior to and during the infection. Data are shown as the mean ± s.d. Asterisks represent statistically significant differences (Student’s t test; **p≤0.005, *p≤0.05).

Our *in vitro* results led us to a model in which GGT acts on GSH to generate Glu and Cys-Gly. These products are then transported into the cytoplasm via GadC and DptA, respectively, where they feed into general metabolism and to the production of cytosolic GSH (Fig 4A). If our model is correct, one would predict that the role of DptA during infection is cysteine acquisition, as has been shown for GGT [13]. Therefore, we tested the ability of a strain lacking DptA to grow in J774 macrophages with and without cysteine supplementation (Fig 4B). As expected based on our Tn-Seq findings, the *dptA* (-) strain displayed a significant intracellular growth defect without cysteine supplementation; however, addition of exogenous cysteine allowed this strain to proliferate to levels indistinguishable from the wild-type (Fig 4B). Taken together with our peptide uptake assays, these findings provide an explanation for our Tn-Seq data; DptA plays a critical role for LVS *in vivo* by transporting Cys-Gly, the breakdown product of host-derived GSH, which the cell requires for protein synthesis.

### Identification of a second GSH-metabolizing enzyme, ChaC

**Fig 5:**
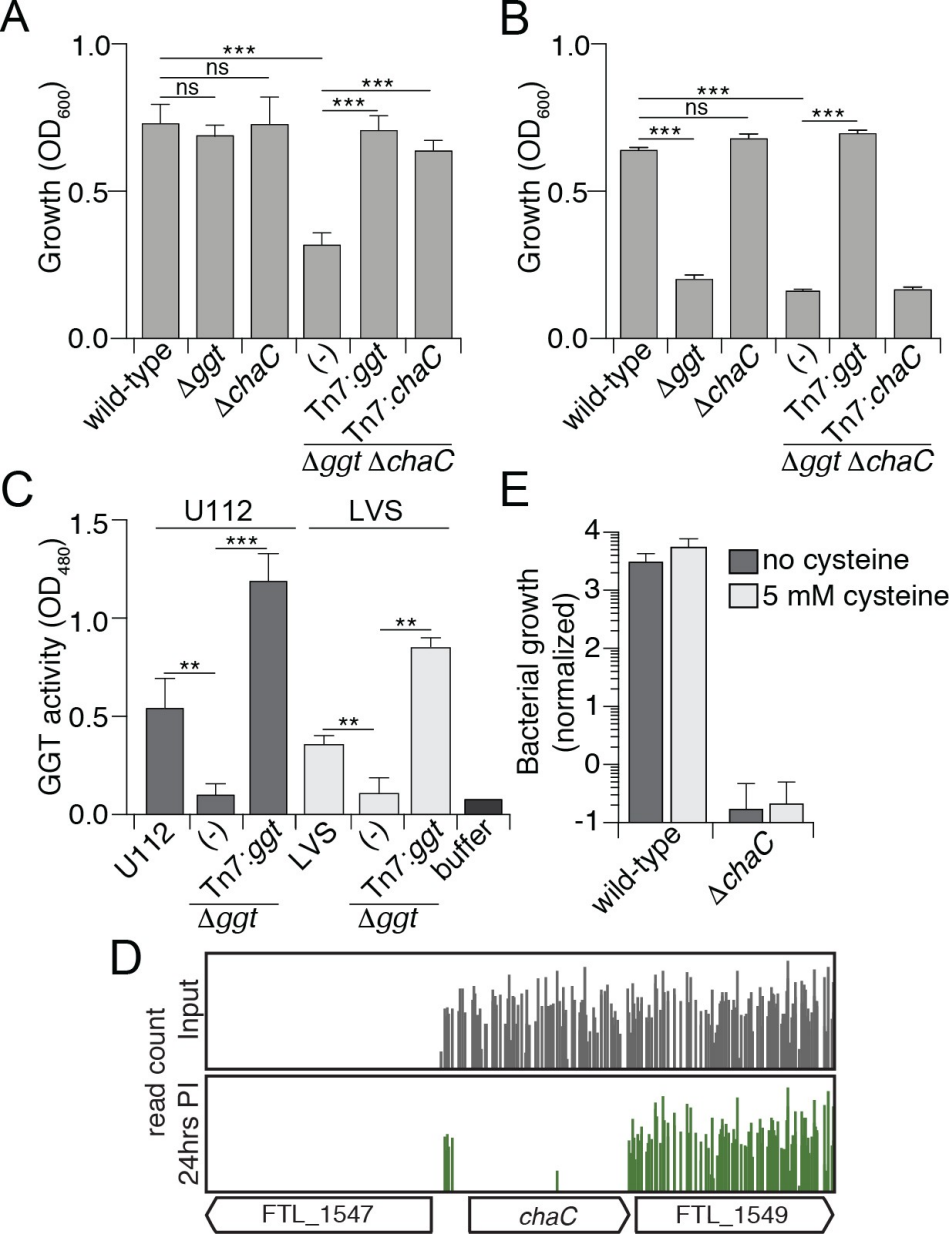
Identification of a second GGT-independent pathway in *Francisella* for GSH catabolism mediated by ChaC. (A-B) OD_600_ measurements of the indicated strains of U112 after 36 hrs of growth in CDM with GSH (A) and γGlu-Cys (B). Data are shown as the mean ± s.d. (C) GGT activity in the indicated strains of U112 and LVS determined via enzyme activity on the substrate L-γ-glutamyl-p-nitroanilide. Data are shown as the mean ± s.d. (D) Transposon insertion profiles from the Tn-Seq screen in J774 cells for the genetic region encompassing *chaC* from the input library (grey) and 24hr post infection (green). Line height represents the relative abundance of sequencing reads at that position on a log scale. (E) Growth of the indicated strains of LVS in J774 cells, normalized to bacterial counts at 2 hrs post infection. J774 cells were either left untreated or treated with 5 mM cysteine prior to and during the infection. Data are shown as the mean ± s.d. Asterisks represent statistically significant differences (Student’s t test; ***p≤0.0005, **p≤0.005, ns, not significant).

In the course of our efforts to understand GSH metabolism in *Francisella*, we tested several mutants within *F*. *tularensis* subsp. *novicida* U112 in parallel with those generated in the LVS background. Surprisingly, we found that–in contrast to our results in LVS–GGT is dispensable for the growth of U112 in media containing GSH as a sole source of cysteine. The U112 Δ*ggt* strain failed to grow on γGlu-Cys as a sole cysteine source, indicating that the GGT protein of this species is synthesized and active (Fig 5A and 5B and S4C and S4D Fig).

One explanation for the capacity of U112 Δ*ggt* to utilize GSH is that cells of this strain encode a second GGT protein. To address this possibility, we compared γ–glutamyl hydrolase activity in cell lysates prepared from wild-type and Δ*ggt* mutant backgrounds of LVS and U112. In both organisms, *ggt* disruption diminished γ–glutamyl hydrolase activity to background levels, suggesting that GGT may be the sole enzyme capable of hydrolyzing γ–glutamyl-containing peptides in these organisms (Fig 5C). However, it is worth noting that the substrate used in this assay, L-γ-glutamyl-p-nitroanilide, is not a natural substrate of GGT and related enzymes. Thus, from these data we could not rule out the possibility that other γ–glutamyl-targeting peptidases are present in LVS and U112. Nevertheless, these findings suggest that U112 possesses multiple pathways for accessing cysteine derived from extracellular GSH.

In eukaryotes, GGT-independent catabolism of cytosolic GSH occurs through the action of γ–glutamyl-cyclotransferase (GGCT) enzymes [45]. This family of proteins acts on a wide range of γ–glutamyl-conjugated peptides, and while the majority of GGCT proteins cannot degrade GSH, one group of these enzymes, those termed ChaC proteins, specifically catabolize GSH into 5-oxoproline and Cys-Gly [17, 46, 47]. ChaC proteins are poorly characterized in prokaryotes; the only such protein from bacteria that has been studied in detail is RipAY, a secreted virulence factor encoded by the plant pathogen *Ralstonia solanacearum* [48, 49]. Therefore, whether ChaC participates in GSH metabolism in bacteria in unclear. Nevertheless, we speculated that a second pathway for catabolism of extracellular GSH in U112 may involve the ChaC protein and searched its genome for sequences that could encode such an enzyme. These sequence searches, in conjunction with *in silico* structural analyses, defined FTN_0599 as the highest probability GGCT-family protein encoded by U112. Henceforth we refer to this protein as ChaC. Notably, ChaC contains an N-terminal transmembrane domain and a prior study demonstrated that the catalytic domain of its ortholog in LVS (98% amino acid identity)– one of the top hits in our Tn-Seq screen (Figs 1C and 5D and S2 Table)–resides in the periplasm and requires DsbA for proper folding [50]. We confirm that, like GGT, ChaC localizes to this compartment and is associated with cellular membranes (S5A and S5B Fig).

We hypothesized that in U112, ChaC accounts for growth in GSH-containing media in the absence of GGT. To test this hypothesis, we generated an in-frame deletion of *chaC* in the wild-type and Δ*ggt* backgrounds of U112 and monitored growth on GSH. As expected, deletions of *ggt* or *chaC* alone had no effect on growth; however, a strain bearing mutations in both genes displayed markedly attenuated growth on GSH as the sole cysteine source. This phenotype was genetically complemented by expressing either gene ectopically from the chromosome (Fig 5A and 5B and S4C and S4D Fig). These findings suggest that in U112, ChaC can liberate GSH-derived species that support *in vitro* growth in media lacking free cysteine. Notably, this does not contradict our γ–glutamyl hydrolase activity assays, as ChaC enzymes are known to act with high specificity and are not predicted to tolerate the non-native substrate L-γ-glutamyl-p-nitroanilide. At this time, we lack an explanation for the differential contribution of ChaC to the *in vitro* growth of U112 and LVS on GSH. Western blot analyses ruled out expression as a simple explanation for our observations (S5C and S5D Fig).

While ChaC is not required for the growth of LVS on GSH *in vitro*, as previously mentioned, ChaC was a top hit in our Tn-Seq screen performed in this strain (Figs 1C and 5D and S2 Table), suggesting this protein is important for growth *in vivo* and that its role is not entirely redundant with GGT. Intramacrophage growth assays confirmed the growth deficiency of LVS Δ*chaC* in this context. However, contrary to our results in the Δ*ggt* strain, exogenous cysteine did not complement the intramacrophage growth defect of LVS Δ*chaC* (Fig 5E). Thus, the role of ChaC within macrophages appears not restricted to generating metabolizable cysteine from GSH. In total, these data highlight the existence of a previously unrecognized GGT-independent pathway capable of the catabolism of GSH by *F*. *tularensis*, but suggest the importance of ChaC in supporting intramacrophage growth extends beyond cysteine acquisition.

## Discussion

Using Tn-Seq we have identified genes that are specifically required for the intramacrophage growth of *F*. *tularensis*. For many of the genes in this catalog the mechanistic basis for their role in intramacrophage growth is poorly understood. Utilization of host-derived GSH is essential for *Francisella* intramacrophage growth and we show that one of the genes from our compendium encodes a peptide transporter that plays a key role in transporting the GSH breakdown product Cys-Gly from the periplasm into the bacterial cytoplasm. In addition, we present evidence that another gene specifies a member of the ChaC family of proteins that is involved in the breakdown of exogenous GSH in *Francisella*.

Our Tn-Seq analyses were conducted with LVS, which is an attenuated derivative of a strain of *F*. *tularensis* subsp. *holarctica*, a type B strain. Recently a Tn-Seq study was performed to define genes essential for rat colonization of the highly virulent type A strain *F*. *tularensis* subsp. *tularensis* SCHU S4 [22]. In principle, this screen should define genes important for multiple aspects of infection, including bacterial uptake, immune evasion, and intracellular proliferation. Nevertheless, comparative analysis between the results obtained in the present study with those generated by Ireland *et al* demonstrate a high degree of overlap, suggesting that many of the genes important for infection in the host are involved in intramacrophage growth. In relation to genes important for the utilization of host-derived GSH, *ggt*, *dptA* and *chaC* were found to play critical roles during infection of the rat with SCHU S4 as assessed by Tn-Seq [22]. Thus, the proteins that play central roles in the metabolism of GSH, which are highly conserved amongst different subspecies of *F*. *tularensis*, are important for the intramacrophage growth of LVS as well as for the fitness of SCHU S4 in the host. We note that GGT has previously been shown to be important for the virulence of both LVS and SCHU S4 in mice [13, 51], and that ChaC (i.e. FTL_1548) has been shown to be important for the virulence of LVS in mice [50].

Many bacteria possess transporters for the uptake of intact exogenous GSH, thereby providing an alternative mechanism for the acquisition of GSH-byproducts [52–54]. Of note, there are no genes that specify predicted GSH transporters present on the genome of *F*. *tularensis* and our *in vitro* findings utilizing ^3^H- and ^35^S-GSH support this observation. Therefore, *F*. *tularensis* relies on proteins present in the periplasm to first degrade GSH, before the resulting breakdown products are transported into the cytoplasm for subsequent further breakdown or utilization (Fig 4A).

Previous work demonstrated a critical role for GGT in the acquisition of cysteine, however, it has remained unclear how the products of this typically periplasmic or extracellular enzyme are transported into the cytoplasm for use in downstream pathways [13, 15]. In fact, in the majority of bacteria it remains unclear how GGT-breakdown products reach the cytoplasm. One exception is the pathogen *Campylobacter jejuni*, which has been demonstrated to transport Cys-Gly into the cytoplasm via the oligopeptide transporter (OPT) CptA [55]. The OPT family of transporters is widespread in bacteria and primarily transports oligopeptides 3-8 a a long. However, homologs of CptA are restricted to a small subset of bacteria found in the oral and intestinal microbiota of mammals. Of note, CptA was found to be non-essential during infection suggesting *C*. *jejuni* possess alternative mechanisms for obtaining the breakdown products of GSH [55].

The results from our saturated Tn-Seq screen led to the identification and characterization of the Cys-Gly transporter, DptA. As a member of the POT family, DptA is powered by the PMF and is capable of transporting multiple di- and tri-peptides with varying affinities. Our results suggest that this protein constitutes the sole mechanism for the transport of Cys-Gly across the inner membrane, making DptA an attractive target for novel therapeutics (Fig 4A). Interestingly, DptA also represents the first member of the POT protein family to be implicated in bacterial pathogenesis.

For many years GGT was believed to be the sole enzyme capable of catabolizing GSH; however, within the last decade, two additional proteins families have been implicated in the breakdown of this molecule. Members of one of these families, termed the Dug2p/3p complex, are restricted to fungi [56, 57] whereas members of the other, ChaC family proteins, belong to the γ-glutamyl cyclotransferase family of proteins and degrade GSH into 5-oxoproline and Cys-Gly [17]. The majority of studies on ChaC proteins have focused on representatives from mice and humans; however, ChaC homologs can be found in both prokaryotes and yeast [16–18]. Multicellular organisms encode two unique copies of ChaC [16]. ChaC1, which participates in the initiation of apoptosis via the unfolded protein response, rapidly catalyzes GSH breakdown *in vitro* (K_cat_ = 225.2±15 min^-1^) [17, 18]. On the other hand, ChaC2 is constitutively expressed and displays much slower GSH turnover (K_cat_ = 15.9± 1 min^-1^). These results led to the hypothesis that ChaC2 functions to catabolize GSH at a low basal rate, whereas ChaC1 functions in response to stress [16]. However, a recent study using an embryonic stem cell model proposed that ChaC2 maintains GSH homeostasis through competitive binding with ChaC1. The authors demonstrate that ChaC2 was capable of inhibiting ChaC1 degradation of GSH in a dose dependent manner, which was important for embryonic stem cell regeneration [58]. Whether this model for the role of ChaC2 is conserved in other cell types was not addressed. Prior to this work, the physiological function of bacterial ChaC proteins had not been investigated; however, *in vitro* studies of *Escherichia coli* ChaC revealed its catalytic properties mirror that of mammalian ChaC2 (K_cat_ = 13±0.27 min^-1^) [16].

Here we identify a ChaC homolog in *F*. *tularensis* and show this protein acts in a GGT-independent pathway to mediate GSH catabolism. To our knowledge, this is the first demonstration that a bacterial ChaC enzyme contributes to the metabolism of GSH. It is unclear why *F*. *tularensis* contains two distinct periplasmic enzymes for the catabolism of GSH. We established cysteine acquisition as the critical function of GGT in intramacrophage growth, yet the intramacrophage growth defect of cells lacking the ChaC enzyme, which can also liberate metabolizable form(s) of cysteine from GSH, cannot be complemented by the addition of exogenous cysteine. Therefore, it could be that ChaC catabolizes GSH, but that cysteine is not the only important product for the cell. Indeed, a study from Charbit and colleagues demonstrated that glutamate acquisition, through the action of the transporter GadC, is essential during infection for defense against reactive oxygen species [39]. The glycine cleavage system is also known to play an important role during murine infection [59]. It is tempting to speculate that ChaC functions to liberate these important metabolites from GSH, perhaps under conditions not conducive to GGT activity, or in a manner coordinated with the machinery necessary for their uptake. However, we cannot rule out the formal possibility that ChaC influences intramacrophage growth in a manner unrelated to its ability to catabolize GSH.

Although this work clearly implicates ChaC in GSH metabolism, precisely how the protein benefits *Francisella* during infection remains to be defined. Our efforts to understand ChaC in *Francisella* may also shed light on the role of the protein in other bacteria. However, bacterial ChaC family proteins are diverse, not restricted to host-associated organisms, and most are not predicted to localize to the periplasm. This suggests that ChaC proteins may serve myriad roles in GSH metabolism idiosyncratic to the lifestyles and physiological needs of bacteria harboring it.

## Materials and methods

### Ethics statement

Animal studies were conducted in compliance with the National Research Council Guide for the Care and Use of Laboratory Animals. All experimental procedures were specifically approved in advance by the University of Washington Institutional Animal Care and Use Committee, under protocol number 2671–06. Animals were group-housed according to experimental group in HEPA-filtered laminar flow cages with cage enrichment and unrestricted access to sterile food and water. The vivarium is managed by the University of Washington Department of Comparative Medicine in compliance with all policies and regulations of the Office of Laboratory Animal Welfare of the Public Health Service. The facility is fully accredited by the American Association for Laboratory Animal Care.

### Bacterial strains and growth conditions

Except where otherwise noted, *F*. *tularensis* subsp. *holarctica* LVS and derivatives were grown aerobically at 37°C in either liquid Mueller-Hinton broth (Difco) supplemented with glucose (0.1%), ferric pyrophosphate (0.025%), and Isovitalex (2%) (MHB) or on cystine heart agar (Difco) supplemented with 1% hemoglobin (CHAH). *F*. *tularensis* subsp. *novicida* strains U112, MFN245 and derivatives were grown aerobically at 37°C in tryptic soy broth or agar supplemented with 0.1% (w/v) cysteine (TSBC or TSAC). *Escherichia coli* strains XL1-blue (Stratagene) or DH5α were used in vector construction. For selection, antibiotics were used at the following concentrations: kanamycin 5 μg mL^-1^ (LVS), 15 μg mL^-1^ (U112) or 50 μg mL^-1^ (*E*. *coli)*, nourseothricin 5 μg mL^-1^, hygromycin 200 μg mL^-1^, and carbenicillin 150 μg mL^-1^.

### Transposon mutant library construction and growth *in vitro*

The mariner transposon delivery plasmid pSD26 [34] was modified to facilitate use of the INSeq protocol [60]. The intermediate plasmid pKL84 was created by amplifying the kanamycin resistance cassette (the *F*. *tularensis* LVS *groES* promoter driving the kanamycin resistance gene [*aphA1*]) from pSD26. The 5´primer included DNA specifying EcoRV and MfeI sites and the 3´primer included DNA specifying NcoI, NheI, and EcoRV sites. The resulting fragment was digested with EcoRV. The mariner transposon delivery plasmid pSAM_Bt [33, 61], which contains MmeI sites within the transposon inverted repeat sequences, was digested with MfeI and XbaI and treated with Klenow large fragment to fill in overhanging 5´ ends. The digested DNAs were ligated together to create the pKL84 plasmid, which contains an MfeI site on the 5´ end of the kanamycin resistance cassette and NcoI, NheI, and the non-unique XbaI sites on the 3´end. To optimize transposase production for LVS, the promoter for *rpoD* of *Bacteroides thetaiotaomicron* was replaced by the 209 bp fragment of DNA upstream of the transposase in pSD26. The promoter-containing fragment was amplified from pSD26 using a 5´ primer that included DNA specifying a BamHI site and a 3´ primer that included DNA specifying an optimal RBS (AGGAGG) followed by a NdeI site and ligated into pKL84 digested with BamHI and NdeI, resulting in the final transposon delivery plasmid pKL91.

To construct the transposon mutant libraries, one microgram of transposon delivery plasmid pKL91 was used to transform *F*. *tularensis* LVS (essentially as described in [62]) in six independent electroporations and cells were plated on CHAH with 5 μg mL^-1^ kanamycin. After two days, the resulting kanamycin-resistant colonies, approximately 800,000, were combined into a single library and frozen. For all Tn-Seq experiments, single aliquots of the library were removed, spread on CHAH with 5 μg mL^-1^ kanamycin, and grown overnight as a confluent lawn. Separate aliquots were grown and used as the inocula for growth in MHB, intramacrophage growth, or to assess mutants viable on solid media. To screen for mutants deficient in growth in liquid media, transposon mutant library cells were diluted to an OD_600_ of 0.005 in 50 mL MHB and grown for ~17 hours (~6.2 doublings) prior to gDNA extraction.

### Transposon mutant library screen in macrophages

Approximately 1x10^7^ of the murine macrophage-like J774A.1 cells were plated on six 150 mm tissue culture plates (~6x10^7^ cells total) in DMEM (Invitrogen) supplemented with 10% fetal bovine serum (Gemini Bio-Products) (DMEM-F) and incubated overnight at 37°C with 5% CO_2_. Macrophage were infected with the LVS transposon mutant library resuspended in DMEM-F at an MOI of approximately 1700. After 2 hours, cells were washed twice with PBS and covered with DMEM containing 10 μg mL^-1^ gentamycin. Cells were incubated at 37°C with 5% CO_2_ for 24 hours then washed twice with PBS and lysed with 1% saponin in PBS for 30 minutes. Lysates were combined and a small aliquot was removed for bacterial enumeration on CHAH. Bacterial cells were pelleted and genomic DNA was directly extracted from pellets (Epicentre MasterPure Complete DNA and RNA Purification Kit).

### INSeq library construction and sequencing

Sequencing libraries were created from 5 samples: (1) input sample for mutants grown in liquid MHB, (2) mutants grown on solid media and not subjected to additional screening, (3) input sample for mutants grown in macrophage (4) output sample for mutants grown in liquid MHB, (5) output sample for mutants grown in macrophage. Libraries were generated from isolated gDNA as in [60], starting with linear PCR using 2 μg DNA per reaction. Libraries were pooled and sequenced using single-end 50 bp sequencing with a single read cluster kit using two lanes of an Illumina HiSeq2500 at 15 pM and 10 pM densities with 20% PhiX and no indexing read. Sequencing reads and processed data files have been deposited at GEO with the accession number GSE138658.

### Tn-Seq data analysis

Sequencing libraries from distinct samples were demultiplexed and trimmed using cutadapt (version 1.16; [63]). Reads were mapped to the LVS genome (NC_007880) using bowtie (version 1.2; [64]) with a seed length of 16 and zero mismatches allowed. Custom scripts were used to identify reads corresponding to insertions in TA sites and tally only those insertions with reads mapping from both ends of the transposon. Datasets were normalized for positional bias and analyzed using EL-ARTIST [35] to identify genes essential for *in vitro* growth using the combined data from all samples grown on solid media. Hidden Markov model analysis resulted in gene classifications (“critical,” “non-essential,” and “both non-essential and critical domains”) after the following sliding window training parameters: 4 TA sites, P < 0.03. CON-ARTIST [35] was used to identify genes critical for growth in liquid media and *in vivo* by comparing inoculum samples to the samples recovered after growth in both MHB and macrophage, respectively. We considered those genes with insertions in at least 4 TA sites, an average fold change of two-fold or more, and with a p-value of less than or equal to 0.05 to be important for survival in a given condition.

### *Francisella* mutant and complementation strain construction

The 3´ end of *dptA* is very close to an *isftu2* repetitive element, making a deletion using allelic exchange difficult. Therefore, we disrupted this gene by plasmid integration. Briefly, the plasmid pEX_*dptA*-frag was created by modification of pKL02 [65], a suicide plasmid which confers resistance to kanamycin. A 441 bp fragment of the *dptA* gene, including DNA specifying a KpnI site, DNA corresponding to amino acids 51–197, a stop codon after amino acid 197, and DNA specifying an EcoRI site, was introduced into pKL02 that had been digested with KpnI and EcoRI. The plasmid pEX_*dptA*-frag was then electroporated into LVS cells and cells in which the plasmid pEX_*dptA*-frag integrated though a single homologous recombination event were selected on CHAH with 5 μg mL^-1^ kanamycin.

In-frame deletion mutations in LVS and U112 were generated via allelic exchange. For LVS, the vector pEX18Kan was employed to make the in-frame deletions of *ggt* and *chaC*, as described previously [66]. To generate deletion vectors for allelic exchange in U112, 1000 bp of flanking regions of *ggt* and *chaC* were cloned into the BamHI and PstI sites of the vector pEX18-pheS-km [8]. U112 deletion strains were then generated as previously described [8]. For all mutants, strains containing the modified genetic region of interested were confirmed by colony PCR and sequencing of PCR products generated from genomic DNA.

The mini-Tn7 system vector pMP749 was utilized for genetic complementation in both LVS and U112 [67]. For *ggt* and *chaC* from LVS, the open reading frame plus the intergenic regions upstream (including the native promoter region) and downstream of the gene were amplified by PCR and cloned into the HindIII and BamHI sites of pMP749. For *dptA*, pMP749 was modified using Gibson assembly to replace the kanamycin resistance cassette for one that encodes nourseothricin resistance. The open reading frame plus the intergenic regions upstream (including the native promoter region) and downstream of *dptA* were amplified by PCR and cloned into the HindIII and BamHI sites of pMP749- nourseothricin. Complementation strains were then generated using these vectors as described in [67]. Briefly, parental strains were initially transformed with the helper plasmid pMP720. Hygromycin resistant colonies were then transformed with the appropriate pMP749-based vector. Kanamycin or nourseothricin resistant colonies were then passage in the absence of hygromycin to cure the bacteria of pMP720. Integration downstream of the *glmS* gene was confirmed by colony PCR. For the complementation of *ggt* and *chaC* in U112, the genes were cloned into pMP749 downstream of the constitutively active *bfr* promoter, and complemented strains were generated as previously described [68]. For localization of ChaC and GGT in U112, the genes encoding *ggt* and *chaC* were cloned downstream of the *bfr* promoter with either a N-terminal VSV-G epitope tag or *phoA* (from *E*. *coli)* gene fusions into the HindIII and BamHI sites of pMP749, and the strains were generated as described above for LVS or U112. As controls for protein localization, full length *phoA* and *phoA* lacking the associated Sec signaling sequence were cloned into the same sites of pMP749. For expression studies in U112 and LVS, the genes encoding *ggt* and *chaC* were cloned with their native promoter regions and a C-terminal VSV-G eptitope tag into the HindIII and BamHI sites of pMP749, and the strains were generated as described above for LVS or U112.

### Mouse infections

Female C57BL/6 mice were purchased from Jackson Laboratories (Bar Harbor, ME) and were 8–10 weeks of age when enrolled in experiments. Mice were exposed to aerosolized bacteria in a whole-body aerosol exposure chamber, as described previously [68, 69]. Briefly, stocks of each bacterial strain were grown to stationary phase at 37°C in supplemented Mueller-Hinton broth diluted in 20% glycerol, aliquoted, and stored at -80°C. For each experiment, aliquots of each strain of bacteria were thawed, streaked onto chocolate agar and incubated at 37°C for 24h. Colonies were harvested and suspended in PBS to a concentration of approximately 3 x 10^9^ CFU/mL, as estimated by optical density and confirmed by quantitative culture. Cohorts of mice were exposed to aerosolized bacteria in a whole animal exposure chamber with a computer interface to control pressures and flows (Biaera Technologies, Hagerstown, MD). Bacterial aerosols were generated by mini-Heart nebulizers with a flow rate of 8 L/min at 40 psi. Dilution air was regulated at 11.5 L/min to maintain total chamber flow at 19.5 L/min during a 10-minute exposure. Actual bacterial deposition was determined by quantitative culture of the homogenized left lungs of four sentinel mice euthanized immediately after completion of the aerosol exposure. The remaining mice were returned to their cages. Mice were observed and weighed daily. At serial time points after infection, mice were euthanized with pentobarbital, exsanguinated by cardiac puncture, and left lungs were harvested and homogenized for quantitative culture on chocolate agar.

### Growth curves

To assess bacterial growth on different cysteine sources, the indicated strains of *Francisella* were grown overnight in Chamberlain’s defined media (CDM) [70] with 1 μM cysteine at 37°C with shaking. After ~16 hrs of growth, the cells were washed three times and resuspended in CDM lacking cysteine. The bacteria were cysteine starved for 2 hrs at 37°C with shaking to allow the cells to metabolize any remaining cysteine. The OD_600_ of the cultures was then measured and the bacteria were diluted to an OD_600_ = 0.1 in CDM with the indicated potential cysteine source at 100 μM. For U112, cultures were transferred in triplicate to a 96-well plate and the OD_600_ of the cultures was monitored for 36 hrs in a plate reader. For LVS, 3 mL cultures inoculated as described above were grown at 37°C and the OD_600_ was determined at the indicated time points.

### GGT activity assays

GGT activity in the indicated strains of LVS and U112 was determined using the γ-Glutamyltransferase (GGT) Activity Colorimetric Assay Kit (Sigma Aldrich). To prepare bacteria for the assay, cells were grown overnight on CDM containing cysteine, then washed three times in CDM lacking cysteine. The OD_600_ of the cells was determined and cultures were normalized to an OD_600_ = 1 (~10^8^ cells) in 1 mL CDM lacking cysteine. Cells were pelleted and resuspended in 100 μL of assay buffer provided in the kit. Cells were lysed by incubating at 50°C for 3 min and cellular debris was removed by centrifugation at max speed for 10 min. The assay was then conducted per the manufacture’s specifications. Activity was measured by reading the absorbance at 480 nM in a plate reader pre-warmed to 37°C.

### GSH uptake assays

The indicated strains of LVS were grown for 16 hrs at 37°C in CDM supplemented with 1 μM cysteine. Cells were spun down, washed three times in uptake buffer (25 mM Tris pH 7.5, 150 mM NaCl, 5 mM glucose), then concentrated 20-fold. The OD_600_ was measured and normalized to OD_600_ = 10. Each reaction contained 20 μL cells, 5μL 100 μM GSH + 0.5 μCi ^3^H-GSH, and 25 μL uptake buffer or competitor substrate at the indicated concentrations (made in uptake buffer). Uptake was allowed to occur for 45 min at 37°C followed by quenching with 1 mL ice cold uptake buffer. Cells were pelleted and washed three times in 1 mL cold uptake buffer, followed by resuspension in 50 μL uptake buffer. Cells were then added to scintillation fluid and counts were measure over 1 minute.

### Macrophage replication assays

Macrophage replication assays for LVS and derivative strains were performed as in [71]. Briefly, approximately 2 x 10^4^ murine macrophage-like J774A.1 cells were infected with LVS or derivative strains at an MOI of 5–10. Culture media containing extracellular bacteria was removed after 2 hours of infection and replaced with DMEM-F containing 10 μg/mL gentamycin. After 2 or 24 hours of infection, macrophage were lysed for 30 minutes in 1% saponin in 1X PBS and plated for enumeration. For assays including cysteine, 5 mM cysteine was added when macrophage were seeded and this concentration was maintained throughout the experiment.

### ^35^S-Cysteine metabolic labeling

The indicated strains of LVS were grown for 16 hrs in CDM, diluted 1:100 into fresh CDM and grown to mid-log phase (OD_600_ = ~0.5–0.7). Cells were then pelleted, washed three times and resuspended in CDM lacking cysteine. The bacteria were cysteine starved for 2 hrs at 37°C with shaking to allow the cells to metabolize any remaining cysteine. The OD_600_ was then normalized to 0.5 in 3 mL cultures of CDM lacking cysteine. Each strain was then supplemented with 100 uM GSH spiked with 5 μCi ^35^S-GSH and grown for 16 hrs at 37°C with shaking. Finally, equal amounts of cells were spun down, resuspended in SDS-PAGE loading buffer, and incubated at 95°C for 10 min. Equal volumes of each sample were loaded in duplicate for separation by SDS-PAGE utilizing an 8–16% SDS-polyacrylamide gels. One set of samples were stained with Coomassie-blue for total protein visualization, while the other half were fixed in 50% methanol, 10% acetic acid and 40% water. The fixed gel was then vacuum-dried and exposed to a phosphoscreen and ^35^S-labeled proteins were visualized on an Azure Sapphire scanner.

### PhoA-activity assays

U112 strains expressing *chaC*- and *ggt-phoA* fusions from the Tn7 insertion site (*attB*), along with several controls (no *phoA*, full length *phoA*, and *phoA* lacking the SecA signal sequence), were grown overnight at 37°C with shaking in CDM media lacking cysteine and supplemented with 100 μM GSH. The OD_600_ of each culture was measured and cells were spun down and resuspended in 1 M Tris-HCl (pH 8.0), 1 mM ZnCl_2_, 0.01% sodium dodecyl sulfate, and 5% chloroform and incubated for 5 min at 37°C to permeabilize cells as previously described [72]. 200 uL of 0.4% *p*-nitrophenyl phosphate dissolved in 1M Tris-HCl pH 8.0 was added to 1 mL permeabilized cells, which were then incubated 8 hrs at 37°C. The resulting mixture was spun down and both the OD_420_ and OD_550_ of the supernatant was determined using a plate reader. The PhoA-activity of each strain was calculated as 1,000 x (OD_420_-OD_550_)/(min xOD_600_ x mL of culture volume).

### Protein localization and expression level measurements

To determine if ChaC is membrane anchored, U112 strains expressing ChaC-VSV-G and ChaC-VSV-G lacking the predicted transmembrane domain (residues 2–22) were grown overnight at 37°C with shaking in CDM. 50 mL of the cultures were pelleted and resuspended in 10 mL 500 mM NaCl_2_, 50 mM Tris pH 7.5 and 10% (v/v) glycerol followed by lysis via sonication. The membrane fraction was separated by centrifuging the lysed cells at 87,207 x g for 2 hrs. The resulting pellet was resuspended in 200 uL of buffer containing 500 mM NaCl_2_, 50 mM Tris pH 7.5 and 10% (v/v) glycerol followed by mixing 1:2 with SDS-PAGE sample loading buffer. The supernatant, corresponding to the soluble fraction, was concentrated 5-fold using a 5 kD cutoff, and was mixed 1:2 with SDS-PAGE sample loading buffer. All samples were then boiled at 95°C for 10 min and processed for Western blotting as described below.

For expression studies, LVS and U112 strains expressing either GGT-VSV-G or ChaC-VSV-G were grown overnight on CDM agar plates. Cells were then back diluted into CDM lacking cysteine supplemented with 100 μM GSH at an OD_600_ = 0.1. Cells were grown to OD_600_ = 0.6–0.8 followed by normalization to OD_600_ = 0.5. Bacterial cell lysates were generated by re-suspending cells in buffer containing 500 mM NaCl_2_, 50 mM Tris pH 7.5 and 10% (v/v) glycerol followed by mixing 1:2 with SDS-PAGE sample loading buffer.

For both expression and protein localization studies, extracted protein samples in SDS-PAGE sample buffer were boiled at 95°C for 10 min and loaded at equal volumes to resolve using SDS-PAGE. Proteins were then transferred onto nitrocellulose membranes which were subsequently blocked in TBST (10 mM Tris-base pH 7.6, 150 mM NaCl_2,_ and 0.1% w/v Tween-20) with 3% (w/v) bovine serum albumin (BSA) for 30 min at 24°C. This was followed by incubation with a monoclonal α–VSV-G antibody (Sigma Aldrich diluted 1:5000), α–Tul4 antibody (BEI diluted 1:3000), α–OpiA antibody ([68] diluted 1:5000) or α–GroEL antibody (diluted 1:15000 provided by Karsten Hazlett, Albany Medical College, Albany, New York, United States) in blocking buffer for 2 hrs at 24°C. Following three washes in TBST, blots were incubated for 45 min in TBST with an α–Rabbit HRP-conjugated secondary antibody (Sigma Aldrich diluted 1:5000) or α–Mouse HRP-conjugated secondary antibody (Millipore diluted 1:5000). The blots were developed using Radiance HRP substrate (Azure Biosystems), visualized using the Azure Biosystems c600, and densitometry analysis was performed using Image J. Density of the proteins corresponding to GGT and ChaC were normalized to the density of the loading control protein GroEL for each sample. Samples were generated and analyzed by Western blot three independent times. Representative images were assembled using Adobe Illustrator CC 2015.

## Supporting information

S1 Fig Characterization of a highly saturated transposon library generated in LVS. (A) HMM analysis using ARTIST [35] identified which of the 2020 annotated genes that are critical, encode critical and non-critical protein regions, or are non-essential *in vitro*. (B) Percentage of potential transposon insertion sites (TA dinucleotides) per gene with a detected transposon insertion for the mutant library grown *in vitro* (blue), the mutant library used to infect macrophage (dark green), and the transposon insertion mutant library recovered after intramacrophage growth (light green). Each bin represents 10% except for the first bin which is 0%.
(PDF)

S2 Fig The purine biosynthesis pathway is critical during macrophage infection by *F*. *tularensis*. The purine biosynthesis pathway of *F*. *tularensis* [11] is depicted with the gene encoding the enzyme responsible for each step listed and colored according to the environment in which they were found to be essential (green, essential *in vitro*; red, dispensable *in vitro* but essential in macrophages).
(PDF)

S3 Fig GGT is predicted to localize to the periplasm in *F*. *tularensis*. Graphical representation of the GGT protein with predicted important features of the protein highlighted. Predictions were based on well-characterized homologs of GGT.
(PDF)

S4 Fig Genes involved in GSH catabolism do not play a role in free cysteine utilization. (A-B) OD_600_ measurements of the indicated strains of LVS after 16 hrs of growth in CDM without (A) or with (B) free cysteine added. (C-D) OD_600_ measurements of the indicated strains of U112 after 36 hrs of growth in CDM without (C) or with (D) free cysteine added. Data in A-D are shown as the mean ± s.d.
(PDF)

S5 Fig GGT and ChaC act within the periplasm and the proteins are expressed in both LVS and U112 under *in vitro* conditions. (A) PhoA-activity of U112 expressing the indicated protein or protein-fusion. Data are shown as the mean ± s.d. of the cumulative results of two biological replicates performed in triplicate. Asterisks represent statistically significant differences (Student’s t test; ***p≤0.0005) (B) Western blot analysis of the localization of ChaC in the indicated strains of U112. Tul4 and OpiA were used as controls for the membrane and soluble fractions respectively. (C) Western blot analysis of ChaC and GGT abundance in the indicated strains of U112 and LVS grown in CDM where GSH is the sole cysteine source. GroEL was utilized as a loading control. (D) Densitometry analysis of ChaC and GGT expression levels from triplicate Western blot analyses as that shown in (C). Data are shown as the mean ± s.d. of three biological replicates.
(PDF)

S1 Table Classification of genes based on EL-ARTIST analysis of *F*. *tularensis* LVS Tn-Seq library mutants grown *in vitro*. (XLSX)

S2 Table Genes identified as important for intramacrophage growth of *F*. *tularensis* LVS using Con-ARTIST analysis. (XLSX)
