# Supplementary figures and images for: Tn-Seq reveals hidden complexity in the utilization of host-derived glutathione in Francisella tularensis
Source: PLoS Pathog. 2020 Jun 3;16(6):e1008566. doi: 10.1371/journal.ppat.1008566 (PMC7340319; doi:10.1371/journal.ppat.1008566)

A

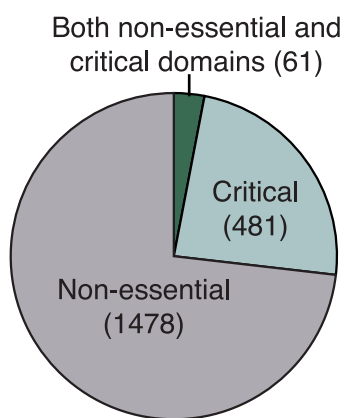

B

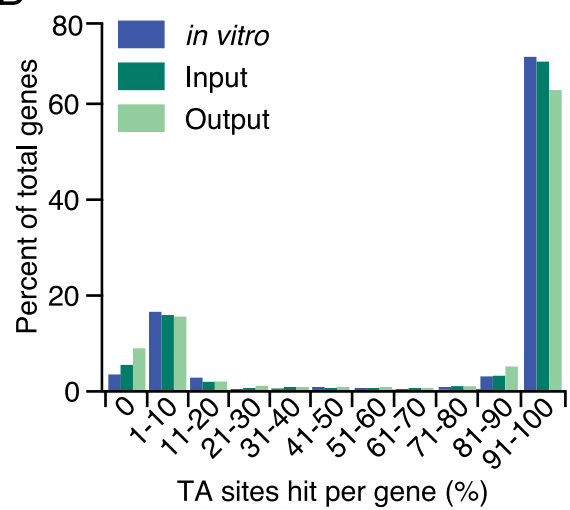

Supplement: S1 Fig — (A) HMM analysis using ARTIST [35] identified which of the 2020 annotated genes that are critical, encode critical and non-critical protein regions, or are non-essential in vitro. (B) Percentage of potential transposon insertion sites (TA dinucleotides) per gene with a detected transposon insertion for the mutant library grown in vitro (blue), the mutant library used to infect macrophage (dark green), and the transposon insertion mutant library recovered after intramacrophage growth (light green). Each bin represents 10% except for the first bin which is 0%. (PDF) [file ppat.1008566.s001.pdf]

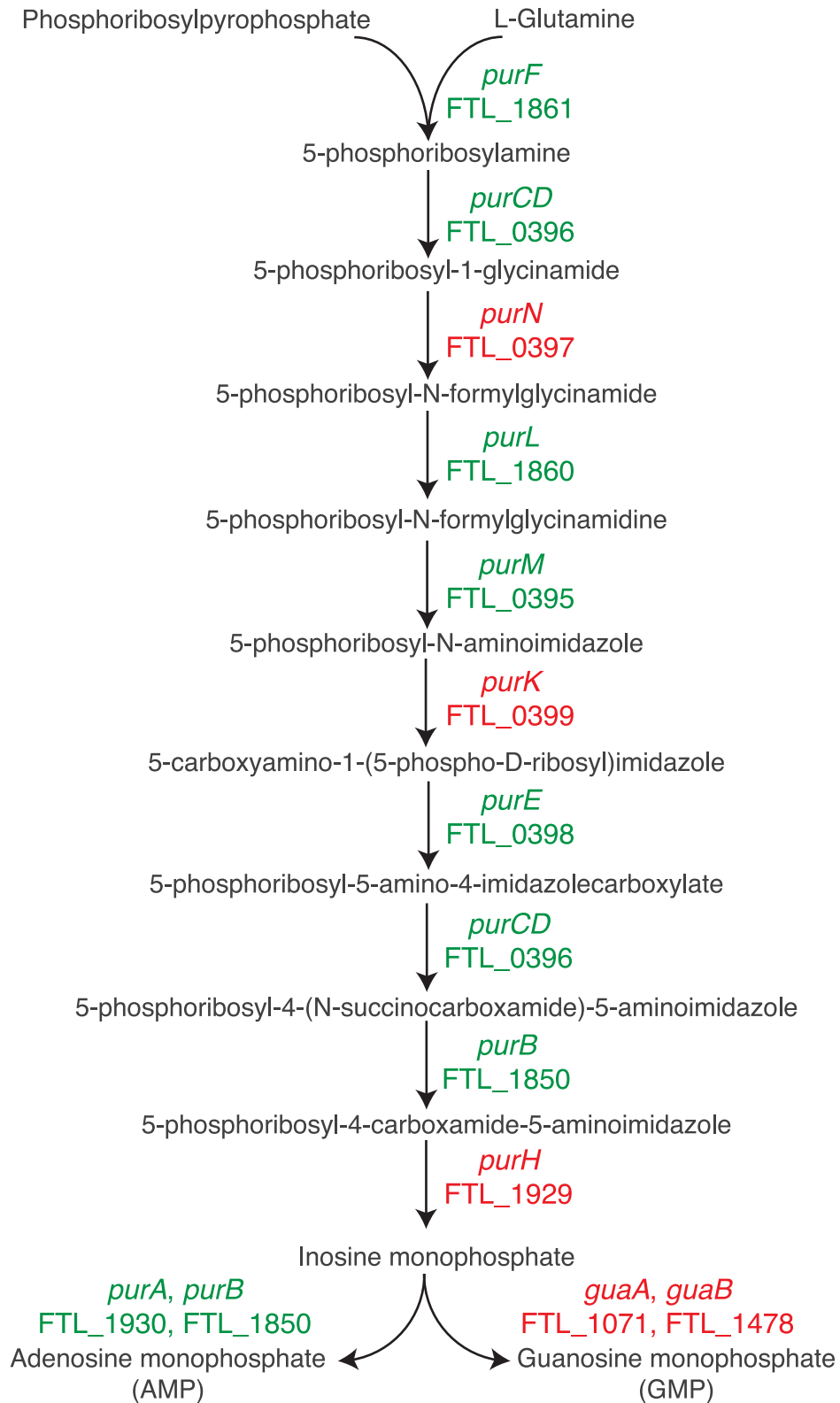

Supplement: S2 Fig — The purine biosynthesis pathway of F. tularensis [11] is depicted with the gene encoding the enzyme responsible for each step listed and colored according to the environment in which they were found to be essential (green, essential in vitro; red, dispensable in vitro but essential in macrophages). (PDF) [file ppat.1008566.s002.pdf]

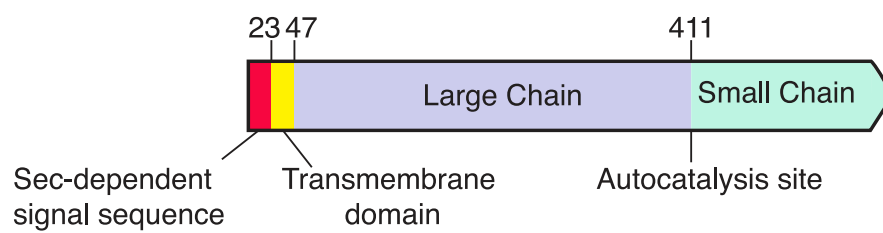

Supplement: S3 Fig — Predictions were based on well-characterized homologs of GGT. (PDF) [file ppat.1008566.s003.pdf]

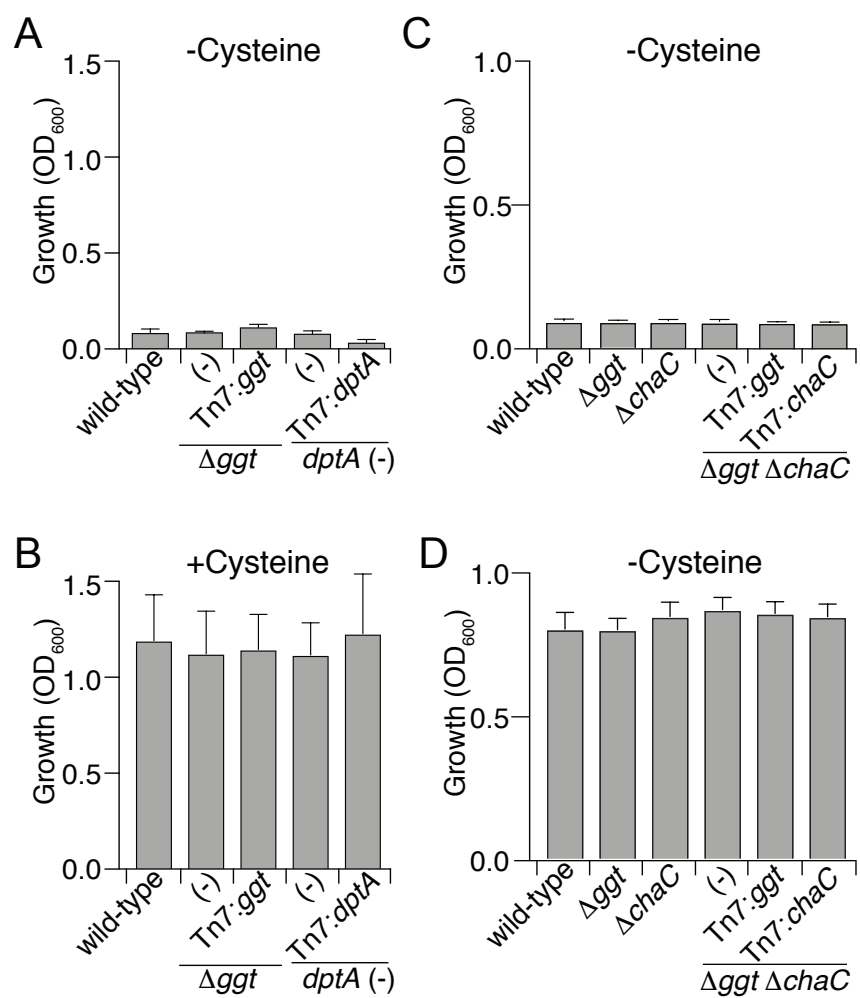

Supplement: S4 Fig — (A-B) OD600 measurements of the indicated strains of LVS after 16 hrs of growth in CDM without (A) or with (B) free cysteine added. (C-D) OD600 measurements of the indicated strains of U112 after 36 hrs of growth in CDM without (C) or with (D) free cysteine added. Data in A-D are shown as the mean ± s.d. (PDF) [file ppat.1008566.s004.pdf]

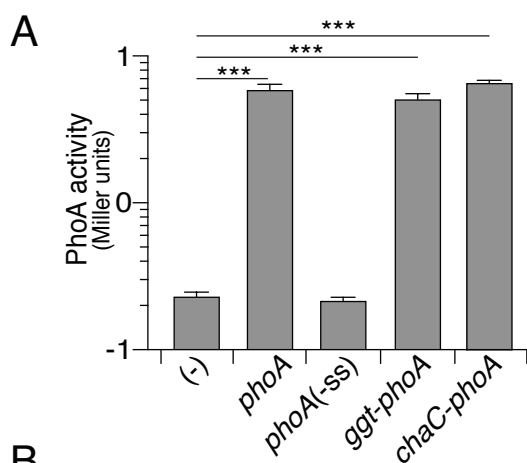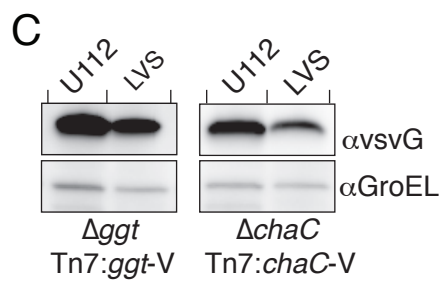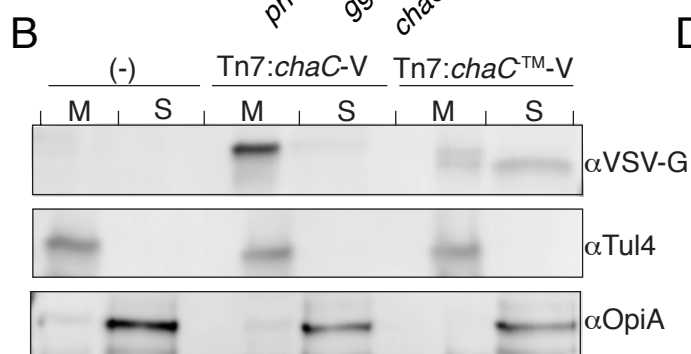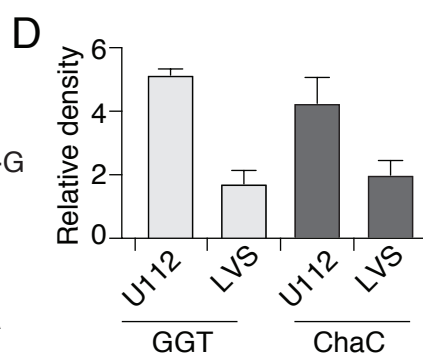

Supplement: S5 Fig — (A) PhoA-activity of U112 expressing the indicated protein or protein-fusion. Data are shown as the mean ± s.d. of the cumulative results of two biological replicates performed in triplicate. Asterisks represent statistically significant differences (Student’s t test; ***p≤0.0005) (B) Western blot analysis of the localization of ChaC in the indicated strains of U112. Tul4 and OpiA were used as controls for the membrane and soluble fractions respectively. (C) Western blot analysis of ChaC and GGT abundance in the indicated strains of U112 and LVS grown in CDM where GSH is the sole cysteine source. GroEL was utilized as a loading control. (D) Densitometry analysis of ChaC and GGT expression levels from triplicate Western blot analyses as that shown in (C). Data are shown as the mean ± s.d. of three biological replicates. (PDF) [file ppat.1008566.s005.pdf]
